# A Systematic Review and Meta‐Analytic Assessment of Unpredictability and Disordered Eating

**DOI:** 10.1111/obr.70022

**Published:** 2025-10-01

**Authors:** Tomás Cabeza de Baca, Hannah T. Fry, Andrés M. Treviño‐Alvarez, Gisela Butera, Brooke Betsuie, Marci E. Gluck

**Affiliations:** ^1^ Obesity and Diabetes Clinical Research Section, Phoenix Epidemiology and Clinical Research Branch, National Institute of Diabetes and Digestive and Kidney Diseases Phoenix Arizona USA; ^2^ Warren Alpert Medical School of Brown University Providence Rhode Island USA; ^3^ Department of Neurology Universidad Autonoma de Nuevo Leon Monterrey México; ^4^ Office of Research Services, Division of Library Services National Institutes of Health Library Bethesda Maryland USA; ^5^ Department of Health Sciences, College of Health and Human Services Northern Arizona University Flagstaff Arizona USA

**Keywords:** appetitive behaviors, childhood, development, life history strategies, life history theory, maladaptive eating behaviors, meta‐analysis, obesity

## Abstract

Perceived unpredictability, whether it relates to experiences, food availability, or belief systems, may predict disordered eating behaviors and affect weight gain and future health. Past studies investigating the associations of unpredictability and disordered eating, however, have shown inconsistent findings. The current review aimed to examine the associations between measures of unpredictability and subjective and objective measures of disordered eating behaviors in adults and children. A systematic review was conducted in July 2023, searching six databases: PubMed/MEDLINE, Embase, Cochrane Library, Web of Science: Core Collection, PsycInfo, and ProQuest Dissertations and Theses, where 20 relevant research articles were identified. Eighty‐three correlation coefficients were extracted from 15 articles (*n* = 9983). Results from a four‐level random effects meta‐analysis found a small, but significant association between unpredictability and disordered eating (*r =* 0.12, 95% CI = 0.08, 0.17, *p* < 0.0001), with a significant (*Q* [82] = 461.55, *p* < 0.0001) and large degree of heterogeneity (*I* ^*2*^ = 86.72%). Country of origin, mode of measurement for disordered eating, chronicity of unpredictability, and study percentage of women were identified as significant moderators. These findings highlight the need for assessment of unpredictability with more rigorous and improved measures of disordered eating to understand the impact on health outcomes.

## 1 Introduction

Obesity is a complex disease, likely due to a combination of genetic predisposition, socioeconomic, cultural, psychological, and behavioral factors. Global rates of obesity have risen to epidemic proportions, with approximately one in two individuals projected to have obesity by 2030, in the USA alone [1]. Obesity is associated with increased risk of diabetes [2], insulin resistance [3, 4], cardiovascular disease [5], and all‐cause mortality [6]. The financial and medical costs associated with complications from obesity are high, as obesity is associated with a 36% increase in annual healthcare costs [7]. Given the financial and health repercussions, it is imperative to identify modifiable factors to intervene that may mitigate the individual risk of obesity.

Traditional models of development and medicine have also noted the importance of understanding the life‐long implications of early adversity, which may relate to unpredictability, on cardiometabolic health [8, 9]. This work suggests that early adversity, broadly, increases obesity risk and cardiometabolic dysfunction [10, 11, 12, 13]. Behavioral mechanisms such as disordered eating or appetitive behavior have been proposed for the linkage between adversity and cardiometabolic health. Indeed, meta‐analyses have specifically found that individuals who report greater experiences of childhood maltreatment and adversity have greater risk of disordered eating [14, 15], particularly with binge eating, bulimia, and obesity [13]. More specifically, researchers have examined eating behaviors more closely, with the majority of studies focusing on overconsumption rather than food restriction [16]. Meta‐analyses and previous research have shown that food insecurity [17] and disordered eating behaviors such as binge eating, susceptibility to hunger, and disinhibition, were associated with increased energy intake and higher BMI [18, 19].

Evolutionary models of development, such as life history theory [20, 21, 22, 23], posit that dimensional aspects of early environmental risk, such as unpredictability (defined as experiences or exposures of inconsistency or unreliability in people, routines, and environment [24]) shape an individual's developmental trajectory and set forth alterations that may result in lifelong implications for health and aging [25, 26]. According to life history theory, children are given a limited amount of time, material, and bioenergetic resources that are strategically allocated toward different developmental facets (behavioral, physiological, psychological) shaped by environmental and household inputs in ways that would improve chances of successfully navigating their adult environment. These developmental “decisions” are instantiated as life history strategies and reflect an individual's pace of life, which resides on a continuum from fast to slow (see [27, 28, 29] for a discussion on measurement of life history strategies) and provide a possible conceptual link between unpredictability and disordered eating [30, 31, 32, 33]. Fast life history strategists are often characterized as having delayed discounting and poorer executive functions such as impulsivity and poor decision making [20, 34, 35], behaviors that are linked to disordered eating [36, 37]. Moreover, early puberty, associated with fast life histories [38, 39], is also positively associated with eating disorders [40, 41]. Eating behavior researchers who incorporate an evolutionary perspective have examined the impact of perceptions of childhood and current unpredictability on appetitive behavior (e.g., [33, 42, 43]), but there are inconsistent findings which may be attributed to study design and methodology. As such, an important step forward is to reconcile these inconsistencies and to pinpoint gaps that future research can address.

### 1.1 Present Study

Given the importance of identifying modifiable and intervenable obesity risk factors and reconciling inconsistent research findings, the main aim of the present study was to perform a systematic review and meta‐analysis of the extant literature to examine the association between perceptions and experiences of unpredictability with disordered eating. Inquiries in environmental unpredictability are often grounded in life history theory (which posits that individuals are given a finite amount of resources that are differentially allocated based on the social and physical environment) and life history strategies (see [27, 28, 44] for a discussion on measurement of life history strategies). As surrogate measures of these are frequently used, subsequent study sub‐aims included (1) examining the association of life history strategy with disordered eating and (2) examining the association of perceptions and experiences of unpredictability with life history strategy on a subset of studies with available, extractable information.

## 2 Method

The study was planned and performed in accordance with the guidelines from the Preferred Reporting Items for Systematic Review and Meta‐Analysis (PRISMA) [45]. The study protocol was registered through PROSPERO [CRD42023453542] (Centre for Reviews and Dissemination, University of York).

### 2.1 Literature Search Strategy

A literature search strategy was developed and conducted in consultation with an experienced research librarian. The search strategy using a combination of keywords and database indexed terms (e.g., Medical Subject Headings [MeSH] and Emtree) was created and underwent an iterative process, including a peer review and piloting phase to ensure the search was comprehensive (see Supplemental Table S1 for an extensive documentation of the literature search keywords). The search was performed on July 18, 2023, from database inception with no date or language limits. Databases searched for relevant literature were PubMed/MEDLINE, Embase, Cochrane Library, Web of Science: Core Collection, and PsycInfo. In addition, we searched ProQuest Dissertations and Theses for gray literature and conducted a citation search of reference lists of included articles.

### 2.2 Inclusion/Exclusion Criteria

Studies eligible for inclusion assessed environmental unpredictability or perceptions of unpredictability using a questionnaire, scale, or through an index (e.g., number of residential changes, school changes, caregiver transitions) and included a measurement of disordered eating or appetitive behavior, assessed using a questionnaire, scale, or index. For the secondary aim, papers that included a psychometric measure of life history strategy were also included. All eligible studies were quantitative, included human participants, and all age groups. Exclusion criteria included non‐human animal studies, no measure of unpredictability or disordered eating/appetitive behavior, and no reviews, theory, commentaries, case studies, editorials, or qualitative analyses. Prior to our screening, multiple reviewers pilot tested eligibility criteria and screening by using a random sample of 25 articles, and we then refined this process.

### 2.3 Screening and Data Extraction

Results from the database searches were initially imported into EndNote 20 reference management software (Clarivate Analytics), with duplicates removed and uploaded to the Covidence systematic review management platform (Covidence.org; Covidence, Melbourne, Victoria, Australia), undergoing an additional de‐duplication of records. Covidence article screening was performed independently by two reviewers (TCdB, HF) using the following three steps: (1) title and abstract screening, (2) full‐text screening, and (3) data extraction. Differences between the two reviewers were discussed and resolved through consensus, with any unresolved conflicts resolved by a third reviewer (AMTA). A data extraction sheet was developed in Excel by three experts and initially piloted. Two reviewers used the standardized coding sheet to extract key study information, sample characteristics (age, sex, race/ethnicity, other sample characteristics [e.g., psychological distress], country of origin of study, BMI, how unpredictability and disordered eating was measured), and relevant statistical parameters (sample size and *r*). Any conflicts were discussed by the two reviewers (TCdB, HF), with a third reviewer resolving lingering conflicts or questions (AMTA). For missing study information not reported in the articles, the study authors were contacted (see below). If relevant variables were coded opposite to our normal extractions (e.g., Mini‐K was coded where higher values = faster life history strategies instead of slower life history strategies), the correlation coefficients were adjusted and mean descriptives were kept the same. Following the completion of article extractions, four reviewers (HF, TCdB, AMTA, MEG) discussed the relevance of the disordered eating constructs.

### 2.4 Quality Assessment

A quality assessment of the risk of bias was assessed through a modified version of the Newcastle–Ottawa Assessment Scale for cohort studies that was designed for use in cross‐sectional studies [46, 47]. The quality assessment tool was developed in consultation with three reviewers who provided feedback and finalized the tool. Once finalized, the tool was piloted, and the results were discussed. For the quality assessment of screened articles, the two screening reviewers provided quality assessments, with a third author discussing and reconciling any incongruities. See Supplemental File S2 for the study quality assessment tool.

### 2.5 Missing Data and Author Contact

First and corresponding authors who had listed email addresses were electronically contacted twice (initial contact and 1‐month follow‐up) for clarification and/or for statistical parameters needed for data extraction. Of the authors contacted for seven articles [31, 42, 43, 48, 49, 50, 51], four authors [31, 43, 48, 51] responded, providing necessary statistical parameters and/or clarification, and the remaining were unresponsive [42, 49, 50]. As a result, one article was dropped for unavailability of extractable information because individual items from the CHAOS measure were used instead of the total score [49], another article provided general linear model *t*‐statistics, which were converted to correlation coefficients [50], and the remaining article was a clarification regarding sex distribution [42]. An author for Salmon et al. [51] provided correlation coefficients on a larger dataset than was reported in the original article, and we used those values for our meta‐analysis.

### 2.6 Power Calculation for Meta‐Analysis

#### 2.6.1 A Priori Power Calculation

An a priori power calculation was performed in the R package *metapower* [52] based on values derived from a prior meta‐analytic pilot study on a subset of five articles. To be conservative, we specified an estimated effect size of 0.20, with an expected sample size of 400 participants per study, and 20 studies/effect sizes, and a large degree of heterogeneity (*I* ^*2*^ = 75%). The fixed‐effects and random‐effects meta‐analysis would both be adequately powered (1.00, 1.00, respectively). An additional analysis was performed to examine whether homogeneity tests would be adequately powered. Given the same parameters, fixed‐effects analyses would be adequately powered to detect effect sizes with high heterogeneity (SD = 5, power = 0.89). Effect sizes with standard deviations ≤ 4 would be underpowered. Given the results of the power analysis, we expected our meta‐analysis to be adequately powered.

#### 2.6.2 Post Hoc Power Calculation

Following completion of data collection and extraction, we recomputed a power calculation based on the updated parameters. Given an estimated effect size of 0.13, with an expected sample size of 361 participants per study, six studies/effect sizes, and a large degree of heterogeneity (*I* ^*2*^ = 86.72%), the fixed‐effects and random‐effects meta‐analysis would both be adequately powered (1.00, 0.90, respectively).

### 2.7 Statistical Analysis

Studies that fit our inclusion criteria were included in our meta‐analysis. Pearson correlation coefficients (*r*) were extracted from study reports. For studies that did not report correlation coefficients, we (1) attempted to contact the study authors (see Missing Data and Author Contact) and (2) attempted to extrapolate correlations based on available parameters.

Two papers did not report the unadjusted correlation coefficients, but included *t*‐statistics from multivariate models, which were converted to correlation coefficients with the following formula [53, 54]:

$$r=\frac{t}{\sqrt{t^{2}+N-2}}$$

Due to many studies reporting several correlation coefficients measured with different measurement tools (e.g., snack taste test, self‐report), all correlation coefficients were kept in the analysis. To account for this, we computed a four‐level random effects meta‐analysis accounting for author/publication and whether articles had more than one study within the published article and estimated statistical significance using *t*‐statistics. All extracted correlation coefficients were converted to Fisher's *Zr* and its associated standard error (SE_*Zr*_) to approximate a normal distribution, and were weighted by inverse variance weights. Results of random effects multilevel meta‐analysis are presented as back‐converted correlation coefficients (*r*) and their associated 95% confidence intervals (95% CI) for each of the study objectives: (1) association between unpredictability and disordered eating, (2) association between life history strategies and disordered eating, and (3) association between unpredictability and life history strategies. Forest plots were created for each of the objectives. An assessment of study heterogeneity was performed with the Q statistic (*p* < 0.05 denotes statistically significant study heterogeneity), and the magnitude of the heterogeneity was quantified by the *I* ^*2*^ index (small, 25%; medium, 50%; large, 75%). A significant degree of heterogeneity was assessed via moderator analyses. Overall moderator model significance (omnibus) was assessed via the Q_m_ statistic (*p* < 0.05 denotes a statistically significant moderator model). Categorical moderator differences were assessed by assigning a reference group to compare to the other groups; continuous moderator analyses were assessed by modeling the associations at relevant values (−1SD, mean, +1SD) if the moderator model was significant. Moderator analyses were performed for the main aim and sub‐aims 1, where moderators were put into individual models. All analyses were performed in *R* statistical software using the *rma.mv* function in the Metafor [55] package using restricted maximum likelihood estimation [56]. An assessment of publication bias was examined visually using a funnel plot and quantitatively via a mixed effects meta‐regression model using the standard error as a predictor. Publication bias assessments were only performed for the main study aim.

## 3 Results

**FIGURE 1:**
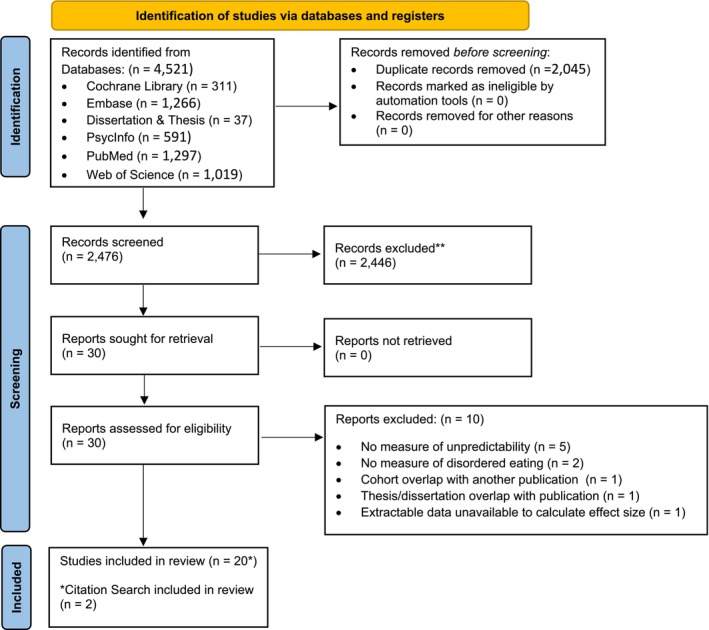
PRISMA diagram.

The initial electronic literature search identified 4521 records. Following the removal of duplicates (k = 2045), 2476 articles were screened for eligibility. Following title and abstract screening, 2446 were deemed irrelevant, with 30 remaining for full text review. After full text review and additional citation search, 20 articles remained with 126 parameters extracted (k = 10 excluded; see Figure 1 for the PRISMA diagram [45] and additional details on the systematic review; also see Supplemental Table S3 for list of excluded articles from full text review).

### 3.1 Study Design

All articles included were published between 2009 and 2024 (unpredictability‐disordered eating range 2014–2024; life history strategies‐disordered eating range 2009–2023). Eleven studies included measures of unpredictability and disordered eating, five studies included measures of life history and disordered eating, and four studies included measures of both unpredictability and life history along with disordered eating. Most studies were conducted in the United States (k = 11), followed by China (k = 6), and Europe (Germany k = 1; Netherlands k = 1; United Kingdom k = 1). The majority of the studies were cross‐sectional (k = 18; longitudinal k = 2). For the sub‐analysis examining life history strategy and disordered eating, the majority of the articles were conducted in China (k = 3), followed by Europe (UK k = 1; Netherlands k = 1, Germany k = 1), and the United States (k = 2).

### 3.2 Sample Characteristics

Most studies included adult samples (k = 11; child sample k = 10; one article had both children and adult samples). A limited number of articles collected and reported data on measures of psychological distress (depression k = 0; anxiety k = 1), glucose values (k = 2; collected but average values not reported), and weight (k = 3). Body mass index (BMI) was reported by 11 articles, both as kg/m^2^ (k = 9) and percentile (k = 1), z‐score (k = 2). Some articles included multiple measures of BMI if there was more than one study or cohort. See Table 1 for study sample characteristics.

**TABLE 1:** Study sample characteristics.

First author last name	Year	Study	Age group	N	Male	Female	Other	Gender missing	BMI mean	BMI measurement	Race/ethnicity
Proffitt Leyva	2020	1	Child	141	66	74			17.56	kg/m^2^	W: 70 A: 8 B: 6 H: 23 O: 13
Abed	2012	1	Adult	206	0	296			22.41	kg/m^2^	W: 193
Appelhans	2014	1	Child	103	49	54			73.6	Percentile	W: 70 A: 8 B: 6 H: 23 O: 13
Doom	2023	1	Child	2587	1255	1332			0.89	Z Score	W: 497 B: 1281 H: 611 O: 199
		2	Child	267	146	121			28.5/30.3	kg/m^2^ (age 32/37)	W: 156 B: 36 O: 75
Eagleton^#^	2022	1	Child	361	180	167		157	NR	NR	W: 438 B: 18 AI/AN: 1 O: 47
Fennis	2022	1	Adult	182	95	87			NR	NR	NR
		2	Adult	278	139	139			NR	NR	NR
		3	Adult	101	52	49			NR	NR	NR
		4	Adult	96	34	62			NR	NR	NR
Ju	2022	1	Child	371	142	154			NR	NR	W: 262 A: 18 B: 16 H: 2 AI/AN: 1 O: 40
Leung	2014	1	Child	380	190	190			NR	NR	W: 212 B: 59 H: 43 O: 63
Luo	2023a	1	Child	85	41	44			1.08	Z Score	A: 85
Luo	2020	1	Child	91	48	43			20.1	kg/m^2^	A: 91
		2	Adult	480	NR	NR			NR	NR	A: 480
Luo	2023b	1	Child	1202	0	1202			18.54	kg/m^2^	A: 1202
			Child	1345	1345	0			19.02	kg/m^2^	A: 1345
Maner	2017	2	Adult	400	188	211	1		26.43	kg/m^2^	W: 311 A: 29 B: 32 H: 21 O: 7
Nettersheim	2018	1	Adult	69	0	69			19.96	kg/m^2^	NR
Proffitt Leyva	2018	1	Adult	353	154	199			NR	NR	NR
		2	Adult	69	16	53			NR	NR	NR
		3	Adult	80	27	53			NR	NR	NR
Salmon^##^	2009	1	Adult	339	0	339			NR	NR	NR
Saltzman	2019	1	Child	108	NR	NR			NR	NR	NR
Ye	2021	1	Adult	3310	1546	1764			NR	NR	A: 3310
Zhou	2023	1	Adult	1010	452	558			NR	NR	A: 1010
Proffitt Leyva	2019	1	Adult	49	26	23			23.43	kg/m^2^	W: 33 A: 3 B: 4 H: 9
Luo	2024	1	Adult	501	NR	NR			20.99	Kg/m^2^	A: 501
			Adult	376	NR	NR			20.99	Kg/m^2^	A: 376

^#^Descriptives are larger than the subset used for correlation. W is white, A is Asian, B is Black, H is Hispanic, AI/AN is American Indian/Alaskan Native, O is Other/multiracial.

^##^Original publication included 100 participants. Upon contacting authors, we were given correlation values for 339 participants. Descriptive values used information from the original 100 participants, and correlation values were from the cohort of 339.

### 3.3 Study Methodology

Unpredictability was measured through a number of scales including the Confusion, Hubbub, and Order Scale (CHAOS; k = 5), Family Unpredictability Scale (FUS; k = 1), Unpredictability Schema Scale (k = 2), Childhood Unpredictability Index (CUI; k = 3), predictability/safety (k = 1), number of household and life transitions (k = 1), and unpredictability composite measures that included multiple unpredictability/harshness scales (k = 5). Some articles had multiple studies with different measures of unpredictability.

Measures of disordered eating varied considerably (see Table 2). The majority of studies measured disordered eating and appetitive behaviors via self‐report (k = 11), while others used parental reports (k = 6) and joint parental–child reports (k = 1). Disordered eating was also measured with objective measures including snack tests (k = 5), food portion choice tasks (k = 2), and food attention bias tasks (k = 1). Some studies had multiple measures of disordered eating behavior. Disordered eating was measured using a multitude of questionnaires and objective tasks that assessed a broad range of eating disorder behavior, from dysregulated eating behaviors (such as food fussiness) to disordered eating, as evaluated through the Eating Disorder Inventory (EDI) [57] and Eating Disorder Examination Questionnaire (EDE‐Q [58]).

**TABLE 2:** Unpredictability, life history, and disordered eating measurements.

First author last name	Year	Study	Unpredictability measure	Life history measure	Eating construct	Eating measure	Eating item mode of measurement
Proffitt Leyva	2020	1	Predictability/Safety	NR	Snack taste test	Eating in absence of hunger (calories consumed)	Snack test
Abed	2012	1	NR	Mini‐K Short Form of the ALHB High‐K Strategy Scale	Disordered eating	EDI‐2 (Garner, 1991) EDE‐Q (Fairburn & Beglin, 2008)	Self‐report
Appelhans	2014	1	CHAOS (Matheny et al., 1995)	NR	Frequency of fruit and vegetable intake Intake of discretionary caloric beverages and fast food Energy‐dense snacks	National Cancer Institutes' Fruit and Vegetable Screener Brief Screener (Nelson & Lytle, 2009) Home Food Inventory (Fulkerson et al., 2008)	Joint parent–child report
Doom	2023	1	Life Transitions 1–5 y, 9–15 y*	NR	Overeating at 9 y (FFCWS)	Parent‐reported CBCL overeating question age 9	Parental report
		2	Life transitions infancy to early childhood, middle childhood to adolescence**	NR	Overeating at 16 y (MLSRA)	Parent‐reported CBCL overeating question age 16	Parental report
Eagleton	2022	1	CHAOS (Matheny et al., 1995)	NR	Food responsiveness, enjoyment of food, emotional overeating, satiety responsiveness, slowness in eating, food fussiness	CEBQ (Wardle et al., 2001)	Parental report
Fennis	2022	1	NR	Mini‐K Short form of the ALHB	Appetitive motivation to acquire hedonic foods	Willingness to pay task (Oliver et al. eating paradigm)	Self‐report
		2	NR	K‐SF‐42	Appetitive motivation to acquire hedonic foods	Willingness to pay task (Oliver et al. eating paradigm)	Self‐report
		3	NR	Mini‐K Short Form of the ALHB	Eating in absence of hunger	Snack taste test (Zellner et al. 2007)	Snack test
		4	NR	Mini‐K Short Form of the ALHB	Eating in absence of hunger‐online	Snack taste test (Zellner et al. 2007)	Snack test
Ju	2022	1	CHAOS (Matheny et al., 1995)	NR	24 months emotional overeating 24 months emotional undereating	Snack taste test (Zellner et al. 2007)	Parental report
Leung	2014	1	CHAOS (Matheny et al., 1995)	NR	Eating in absence of hunger (calories consumed) Food responsiveness, enjoyment of food, emotional overeating, satiety responsiveness Tantrums over food	Snack taste test (Birch et al., 2003) CEBQ (Wardle et al., 2001) Agras et al. 2004	Snack test Parental report Parental Report
Luo	2023a	1	FUS (Ross & Hill, 2000); CUI (Mittal et al., 2015); Subjective SES (Mittal et al., 2015)	NR	Food responsiveness, enjoyment of food High calorie food portion, low calorie food portion	CEBQ (Wardle et al., 2001) Food portion choice task (Sim et al., 2018)	Parental report Food portion choice task
Luo	2020	1	CUI (Mittal et al., 2015); FUS (Ross & Hill, 2000)	NR	High calorie food portion, low calorie food portion	Food portion choice task (Sim et al., 2018)	Food portion choice task
		2	CUI (Mittal et al., 2015); FUS (Ross & Hill, 2000)	Mini‐K	Overeating	Loss of control eating subscale (Angle et al., 2009)	Self‐report
Luo	2023b	1	FUS (Ross & Hill, 2000); CUI (Mittal et al., 2015); Subjective SES (Mittal et al., 2015)	Mini‐K	Overeating	Loss of control subscale of TFEQ (Anglé et al., 2009)	Self‐report
Maner	2017	2	CUI (Mittal et al., 2015)	Mini‐K	Dysregulated eating behavior	Dysregulated eating behavior	Self‐report
Nettersheim	2018	1	NR	Arizona Life History Battery (ALHB)	Disordered eating	EDE‐Q (Fairburn & Beglin, 2008)	Self‐report
Proffitt Leyva	2018	1	USS (Proffitt Leyva & Hill, 2018; Cabeza de Baca et al., 2016) CUI (Mittal et al., 2015)	NR	Body awareness	BAQ (Shields, Mallory, & Simon, 1989)	Self‐report
		2	USS (Proffitt Leyva & Hill, 2018; Cabeza de Baca et al., 2016)	NR	Body awareness Mindful eating eating in absence of hunger	BAQ (Shields, Mallory, & Simon, 1989) Mindful Eating (Framson et al., 2009) EAH‐A (Tanofsky‐Kraff et a., 2008)	Self‐report
		3	USS (Proffitt Leyva & Hill, 2018; Cabeza de Baca et al., 2016)	NR	Eating in absence of hunger (calories consumed) Body awareness	Snack taste test BAQ (Shields, Mallory, & Simon, 1989)	Snack test Self‐report
Salmon	2009	1	NR	Arizona Life History Battery (ALHB)	Disordered eating	EDI‐2 (Garner, 1991)	Self‐report
Saltzman	2019	1	CHAOS (Matheny et al., 1995)	NR	Food responsiveness, emotional overeating, enjoyment of food, satiety responsiveness, slowness in eating, emotional undereating, food fussiness	CEBQ (Wardle et al., 2001)	Parental report
Ye	2021	1	NR	Mini‐K	Overeating	Loss of control subscale of TFEQ (Anglé et al., 2009)	Self‐report
Zhou	2023	1	CUI (Zhou et al., 2018)	Mini‐K	Food‐addiction	YFA 2.0 (Schulte & Gearhardt, 2017)	Self‐report
Proffitt Leyva	2019	1	USS (Proffitt Leyva & Hill, 2018; Cabeza de Baca et al., 2016) CUI (Mittal et al., 2015; Mittal & Griskeveious 2014) FUS (Ross & Hill, 2000)	NR	Snack taste test	Eating in absence of hunger (calories consumed)	Snack test
Luo	2024	1	FUS (Ross & Hill, 2000); CUI (Mittal et al., 2015); Subjective SES (Mittal et al., 2015)	NR	Binge eating tendencies	Loss of control subscale of TFEQ (Anglé et al., 2009)	Self‐report
			FUS (Ross & Hill, 2000); CUI (Mittal et al., 2015); Subjective SES (Mittal et al., 2015)	NR	Food attention bias task	Food attention bias to high calorie foods, food attention bias to low calorie foods	Food attention bias task

* Changes in residence, changes in parental cohabitation, changes in employment, and changes in father's incarceration.

** Changes in residence, changes in cohabitation, and changes in employment.

Measurement of life history strategies predominately used the Mini‐K scale [34] (k = 7), followed by the High‐K Strategy Scale [59] (HKSS; k = 1), and the Arizona Life History Battery [60] (ALHB; k = 2), K‐SF‐42 [61] (k = 1). These scales and battery are scored such that higher scores denote slower life history strategies, which are characterized by more prosocial and enduring relationships, greater planning, insight, and control.

### 3.4 Quality Assessment

Quality assessment for the studies included in the meta‐analysis ranged from 6 to 10 on a scale from 0 to 10 (*M =* 8.30, SD = 1.08; see Table 3 for quality assessment; see Supplemental File S2 for study quality assessment tool).

**TABLE 3:** Quality assessment.

		Max 1 star	Max 1 star	Max 1 star	Max 3 stars	Max 2 stars	Max 2 stars	Max 1 star	Max 10 pts
First author last name	Year	SELECTION 1	SELECTION 2	SELECTION 3	SELECTION 4	COMPARABILITY 1	OUTCOME 1	OUTCOME 2	Total
Proffitt Leyva	2020	*	0	0	*	**	**	*	**7**
Abed	2012	*	0	0	**	0	**	*	**6**
Appelhans	2014	*	0	*	**	**	**	*	**9**
Doom	2023	*	0	*	*	**	*	*	**7**
Eagleton	2022	*	0	*	**	**	**	*	**9**
Fennis	2022	0	*	*	**	**	*	*	**8**
Ju	2022	*	0	*	**	**	**	*	**9**
Leung	2014	*	0	0	**	**	**	*	**8**
Luo	2023a	0	0	*	**	**	**	*	**8**
Luo	2020	*	*	*	**	**	**	*	**10**
Luo	2023b	*	0	*	**	**	**	*	**9**
Maner et al	2017	0	0	0	**	**	*	*	**6**
Nettersheim	2018	*	0	0	**	**	**	*	**8**
Proffitt Leyva	2018	0	*	*	**	**	**	*	**9**
Salmon	2009	0	*	0	**	**	**	*	**8**
Saltzman	2019	*	0	*	**	**	**	*	**9**
Ye et al	2021	*	*	0	**	**	**	*	**9**
Zhou	2023	*	0	*	**	**	**	*	**9**
Proffitt Leyva	2019	0	*	*	**	**	**	*	**9**
Luo	2024	*	0	*	**	**	**	*	**9**

### 3.5 Meta‐Analysis: Unpredictability and Disordered Eating

**FIGURE 2:**
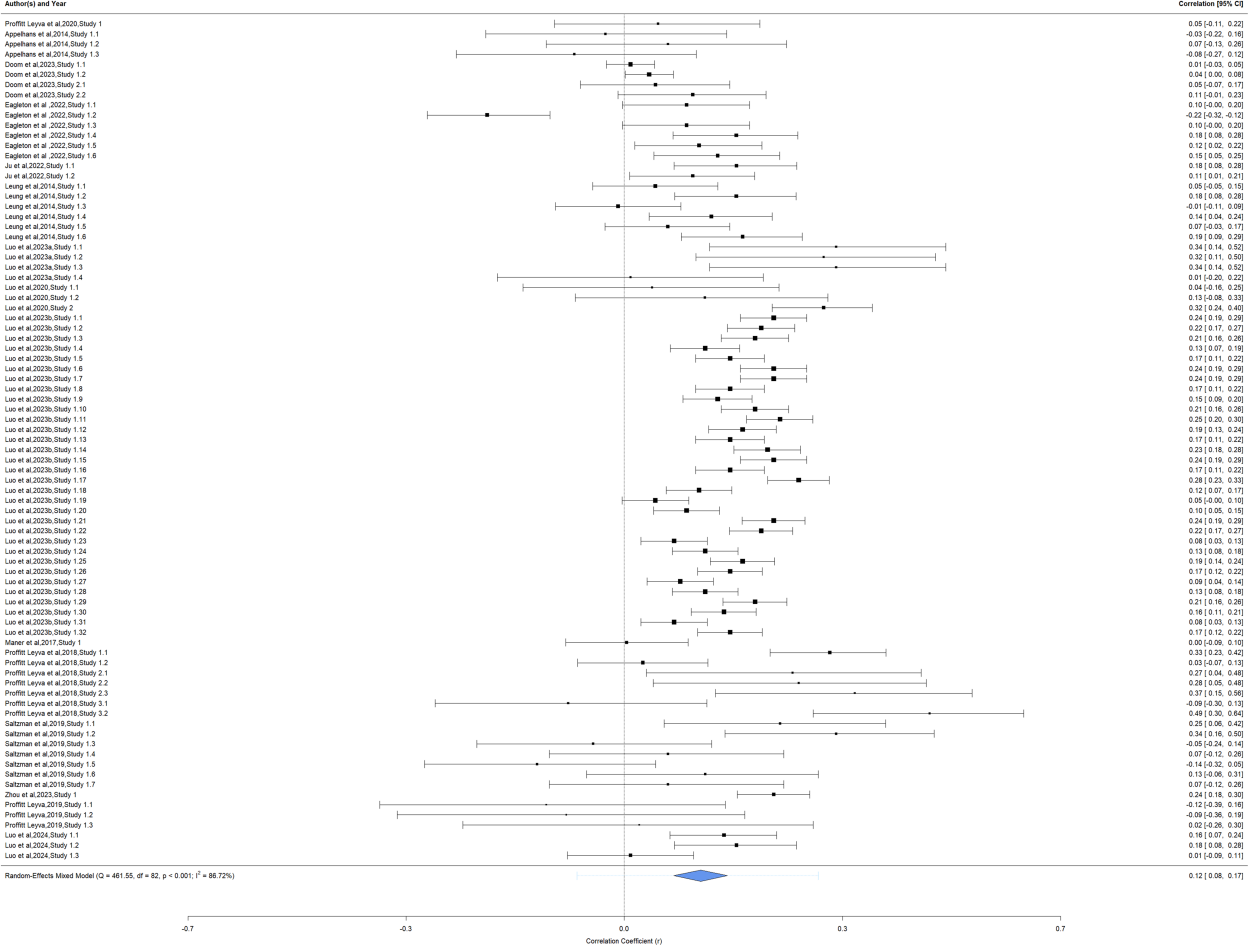
Forest plot of unpredictability and disordered eating. Meta‐analytic results of child and adult studies examining the association between unpredictability and disordered eating. The forest plot presents the extracted correlation coefficients (*r*) and 95% confidence intervals (95% CI) for the studies organized by first author and year, study number, and the number of extracted parameters of each study. Larger studies were denoted by larger correlation boxes. The meta‐analytic result is represented by a blue diamond.

Fifteen articles with 83 extracted correlations and sample sizes (*n =* 9983) were included in the analysis (see Figure 2 for forest plot). The association between unpredictability and disordered eating was small, positive, and significant (*r =* 0.12, 95% CI = 0.08, 0.17, *p* < 0.0001). A significant (*Q* [82] = 461.55, *p* < 0.0001) and large degree of heterogeneity (*I* ^*2*^ = 86.72%) was identified. The heterogeneity occurred mostly between correlation coefficients (50.37%) followed by between articles (36.22%) and between studies within articles (0.13%).

A sensitivity analysis was performed on a subset of parameter estimates derived from cross‐sectional analyses, and there were no major changes to the magnitude or patterning of results (*r =* 0.13, 95% CI = 0.08, 0.18, *p* < 0.0001), but there was a slight reduction in heterogeneity (*I* ^*2*^ = 84.01%). A longitudinal‐only analysis revealed an attenuated non‐significant correlation (*r =* 0.11, 95% CI = −0.02, 0.23, *p* = 0.10) with a significant and large amount of heterogeneity (*I* ^*2*^ = 92.56%). A final sensitivity analysis was performed that removed longitudinal associations that were contrary to the original hypotheses (e.g., remove correlations where unpredictability assessments came after disordered eating; *r* = 0.10, 95% CI = −0.01, 0.21, *p* = 0.08; *I* ^*2*^ = 90.58%).

### 3.6 Moderator Analyses

Country study (China/USA), age group status (Adult/Child), mode of measurement for disordered eating, chronology of unpredictability (Childhood Unpredictability/Adult Unpredictability), study percentage of women, and study percentage of white participants were separately assessed as potential moderators (see Table 4 for complete results). A priori analysis outlined assessing other characteristics such as glucose, psychological distress, and measures of adiposity (see above for an explanation), but few studies reported data on these characteristics, and these moderators were not assessed.

**TABLE 4:** Moderator analyses for unpredictability and disordered eating.

Unpredictability and disordered eating				
Country of origin (Q_M_ [1, 81] = 7.76, *p* = 0.0067) *I* ^*2*^ *=* 83.16%				
	*r*	95% CI		*p*
USA [reference]	0.09	0.04	0.13	
China	0.19	0.13	0.25	0.0067
**Age group (Q_M_ [1, 81] = 1.48, *p* = 0.23) *I* ^*2*^ *=* 86.85%**				
	***r***	**95% CI**		***p***
Adult [reference]	0.16	0.08	0.24	
Child	0.10	0.05	0.16	0.23
**Mode of measurement (Q_M_ [5, 77] = 3.79, *p* = 0.004) *I* ^*2*^ *=* 82.26%**				
	***r***	**95% CI**		***p***
Self‐report [reference]	0.20	0.15	0.25	
Food attention bias task	0.10	−0.04	0.24	0.18
Food portion choice task	0.11	−0.03	0.24	0.20
Joint parent–child report	−0.01	−0.17	0.14	0.01
Parental report	0.10	0.05	0.15	0.0075
Snack taste TEST	−0.01	−0.12	0.10	0.0007
**Mode of measurement (Q_M_ [1, 81] = 2.90, *p* = 0.09) *I* ^*2*^ *=* 86.14%**				
	***r***	**95% CI**		***p***
Objective [reference]	0.05	−0.05	0.15	
Subjective	0.13	0.09	0.18	0.09
**Chronology of unpredictability (Q_M_ [1, 81] = 5.22, *p* = 0.03) *I* ^*2*^ *=* 85.69%**				
Childhood [Reference]	0.11	0.07	0.15	
Adulthood	0.26	0.13	0.37	0.03
**Study percentage of women (Q_M_ [1, 70] = 4.88 *p* = 0.03) *I* ^*2*^ *=* 87.65%**				
	***r***	**95% CI**		***p***
−1 SD Percentage (16%)	0.10	0.04	0.15	0.0005
Mean Percentage (51%)	0.11	0.06	0.17	0.03
+1 SD Percentage (86%)	0.13	0.08	0.19	< 0.00001
**Study percentage of white participants (Q_M_ [1, 67] = 3.39, *p* = 0.07) *I* ^*2*^ *=* 86.90%**				

*Note:* For continuous moderators (i.e., study percentage of women), *p*‐values were calculated via conversion of 95% CIs to z‐scores.

#### 3.6.1 Country of Origin

Country of origin was a significant moderator (*Q*_*M*_ [1, 81] = 7.76, *p* = 0.0067). Studies that were conducted in China (k = 5; *n* = 4714) had a significantly higher association (*r* = 0.19, 95% CI = 0.13, 0.25) in comparison to the United States (k = 10, *n* = 5120; *r* = 0.09, 95% CI = 0.04, 0.13).

#### 3.6.2 Age Group Status

Group status was not a significant moderator (*Q*_*M*_ [1, 81] = 1.48, *p* = 0.23). Statistically, studies with adult samples (k = 6; *n* = 2942) did not differ from studies with child samples (k = 10; *n* = 7041).

#### 3.6.3 Mode of Measurement

Mode of measurement was a significant moderator (*Q*_*M*_ [5, 77] = 3.79, *p* = 0.004; see Table 2 for the two modes (Objective vs. Subjective) of measurement results). Using self‐report as a reference (k = 6; *n* = 7740; *r* = 0.20, 95% CI = 0.15, 0.25), joint parent–child reports (k = 1, *n* = 103; *r* = −0.01, 95% CI = −0.17, 0.14), parental reports (k = 6, *n* = 4159; *r* = 0.10, 95% CI = 0.05, 0.15), and the snack taste test (k = 4, *n* = 650; *r* = −0.01, 95% CI = −0.12, 0.10) were significantly different. There were no significant differences between self‐reported measures of disordered eating, food attention bias task (k = 1; *n* = 376), and food portion choice task (k = 2; *n* = 176; see Table 2).

A sensitivity analysis was performed where mode of measurement and country of origin were entered as simultaneous moderators to examine whether the country effect was driven by how disordered eating was collected in the countries (i.e., all snack taste tests were collected in the USA and self‐report was the predominate mode for China). The model was significant (*Q*_*M*_ [6, 76] = 3.08, *p* = 0.001) and the differences between China and the USA were no longer significant (*p* = 0.17), but the difference between self‐report and snack test remained (*p* = 0.02).

#### 3.6.4 Chronology of Unpredictability

Because it is essential to differentiate between the developmental effects of childhood unpredictability and the proximate effects of current unpredictability or perceptions of unpredictability, we classified and coded extracted unpredictability measures on whether they assessed unpredictability experienced or perceived in childhood or during adulthood. Childhood measures we classified as such if they were retrospective (i.e., asked adults to report unpredictability in childhood such as the CUI or retro‐FUS), were filled out by a parent about a child's experience, or were filled out by a child/adolescent (e.g., CHAOS). Measures that assessed perceptions of unpredictability in adulthood (e.g., USS) were classified as adult. Source of unpredictability was a significant moderator (*Q*_*M*_ [1, 81] = 5.22, *p* = 0.03). Using childhood as a reference (k = 15; *n* = 10,210; *r* = 0.11, 95% CI = 0.07, 0.15), adult unpredictability was significantly higher in comparison (k = 2; *n* = 551; *r* = 0.26, 95% CI = 0.13, 0.37).

#### 3.6.5 Study Percentage of Women

Study percentage of women (k = 13; female *n* = 4478) was found to be a statistically significant moderator (*Q*_*M*_ [1, 70] = 4.88, *p* = 0.03). The association was disaggregated at −1 SD (16%), Mean (51%), and +1 SD (86%). As shown in Table 2, the patterning suggests that the larger the percentage of women in the study sample, the stronger the association between unpredictability and disordered eating.

#### 3.6.6 Study Percentage of White Participants

There was no significant moderation by study percentage of white participants (k = 13 report White demographics; *n* = 1981) (*Q*_*M*_ [1, 67] = 3.39, *p* = 0.07).

### 3.7 Publication Bias

Visual inspection of the funnel plots (Supplemental Figure S4) showed that the majority of the studies are at the top right of the funnel; however, there was no evidence for asymmetry (*z =* −1.42, *p* = 0.16).

### 3.8 Meta‐Analysis: Life History Strategy and Disordered Eating

**FIGURE 3:**
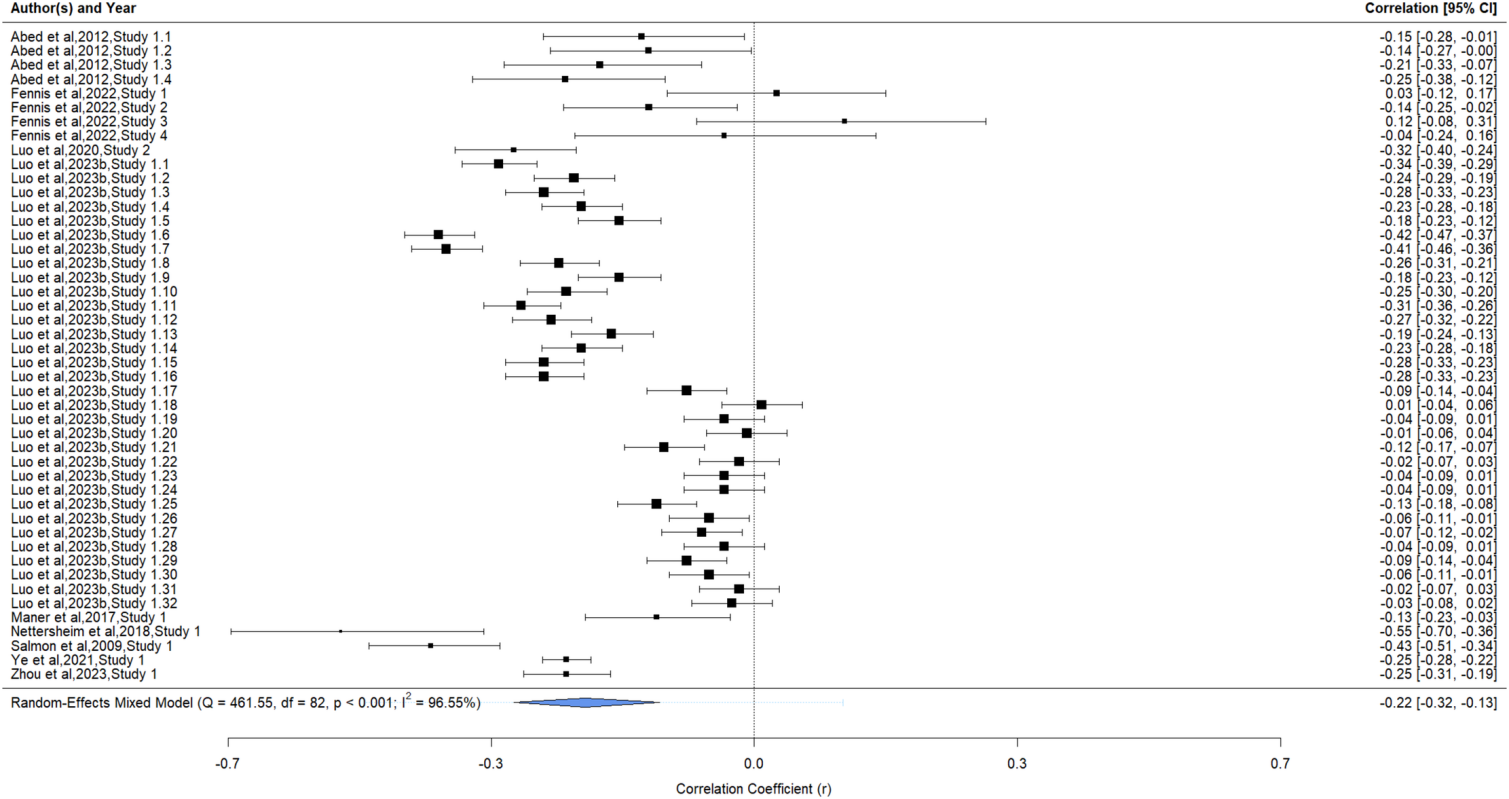
Forest plot of slower life history strategies and disordered eating. Meta‐analytic results of child and adult studies examining the association between slower life history strategies and disordered eating. The forest plot presents the extracted correlation coefficients (*r*) and 95% confidence intervals (95% CI) for the studies organized by first author and year, study number, and the number of extracted parameters of each study. Larger studies were denoted by larger correlation boxes. The meta‐analytic result is represented by a blue diamond.

The analysis examining the association between life history strategy and disordered eating included nine articles with 46 extracted correlations and sample sizes (*n =* 9018; see Figure 3 for forest plot). The association was small, negative, and significant (*r =* −0.22, 95% CI = −0.32, −0.13, *p* < 0.0001). A significant (*Q* [45] = 774.30, *p* < 0.0001) and large degree of heterogeneity (*I* ^*2*^ = 96.55%) was identified. The heterogeneity occurred mostly between correlation coefficients (52.45%), followed by between articles (44.11%) and between studies within articles (0.00%).

#### 3.8.1 Country of Origin

All countries located in Europe were aggregated together for the analysis (see Table 5 for moderation analysis by all countries). There was no significant moderation by country of origin (Q_M_ [2, 43] = 0.14, *p* = 0.87).

**TABLE 5:** Moderator analyses for slower life history strategies and disordered eating.

Slower life history strategies and disordered eating				
Country of origin (Q_M_ [4, 41] = 2.42, *p* = 0.06) *I* ^*2*^ *=* 95.37%				
	*r*	95% CI		*p*
USA [reference]	−0.29	−0.46	−0.08	
China	−0.21	−0.30	−0.11	0.48
Germany	−0.55	−0.76	−0.24	0.13
Netherlands	−0.02	−0.21	0.18	0.06
UK	−0.19	−0.36	0.00	0.46
**Country of origin (Q_M_ [2, 43] = 0.14, *p* = 0.87) *I* ^*2*^ *=* 97.29%**				
	***r***	**95% CI**		***p***
USA [reference]	−0.29	−0.51	−0.02	
China	−0.23	−0.39	−0.07	0.72
Europe	−0.20	−0.38	−0.01	0.6
**Age group (Q_M_ [1, 44] = 0.32, *p* = 0.57) *I* ^*2*^ *=* 97.05%**				
	***r***	**95% CI**		***p***
Adult [reference]	−0.25	−0.36	−0.12	
Child	−0.17	−0.41	0.11	0.57
**Mode of measurement (QM [1, 44] = 2.54, *p* = 0.12) *I* ^*2*^ *=* 95.59%**				
	*r*	95% CI		*p*
Self‐report [reference]	−0.22	−0.30	−0.14	
Snack taste test	−0.02	−0.27	0.24	0.12
**Study percentage of women (QM [1, 43] = 114.96, *p* < 0.001) *I* ^*2*^ *=* 94.06%**				
	***r***	**95% CI**		***p***
Minus 1 SD percentage (11%)	−0.10	−0.19	−0.01	0.03
Mean percentage (57%)	−0.20	−0.29	−0.12	< 0.00001
Max percentage (100%)	−0.30	−0.38	−0.21	< 0.00001
**Study percentage of white participants (QM [1, 38] = 0.01, *p* = 0.91) *I* ^*2*^ *=* 94.72%**				

*Note:* For continuous moderators (i.e., study percentage of women), *p*‐values were calculated via conversion of 95% CIs to z‐scores. Maximum percentage (100%) was used because +1 SD exceeded possible value.

#### 3.8.2 Age Group Status

There was no significant moderation by age group status (Q_M_ [1, 44] = 0.32, *p* = 0.57).

#### 3.8.3 Mode of Measurement

Studies that included measured life history strategies used only self‐report or snack taste test as measures of disordered eating. There was no significant moderation by mode of measurement (Q_M_ [1, 44] = 2.54, *p* = 0.12).

#### 3.8.4 Study Percentage of Women

Study percentage of women (k = 84, 686; female *n* = 4686) was found to be a statistically significant moderator (Q_M_ [1, 43] = 114.96, *p <* 0.001). The association was disaggregated at −1 SD (11%), mean (57%), and the maximum potential percentage (100%). As shown in Table 5, the patterning suggests that the larger the percentage of women in the study sample, the stronger the association between slower life history strategies and disordered eating.

#### 3.8.5 Study Percentage of White Participants

There was no significant moderation by study percentage of white participants (Q_M_ [2, 43] = 0.14, *p* = 0.87).

### 3.9 Meta‐Analysis: Unpredictability and Life History Strategy

**FIGURE 4:**
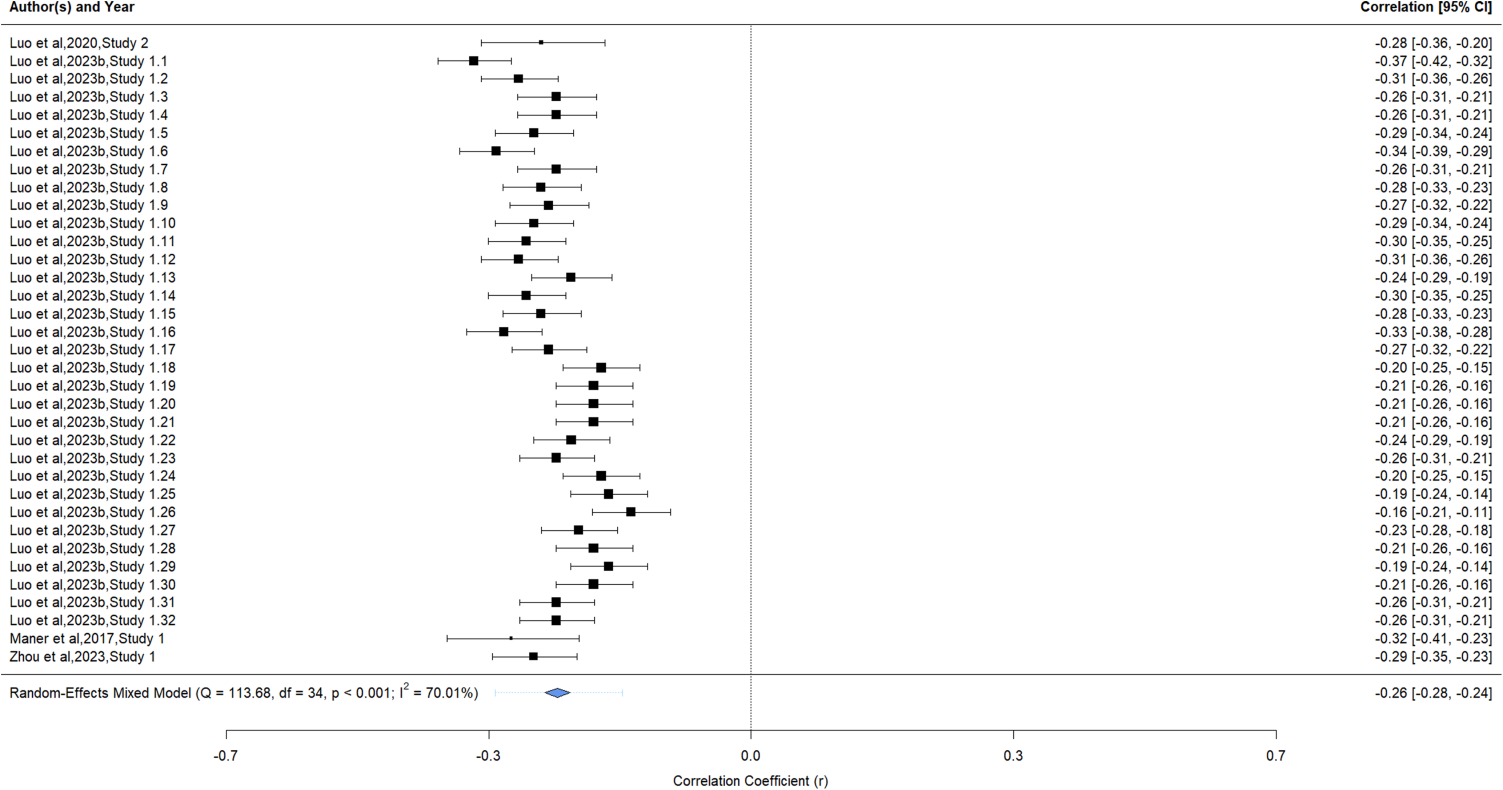
Forest plot of unpredictability and slower life history strategies. Meta‐analytic results of child and adult studies examining the association between unpredictability and slower life history strategies. The forest plot presents the extracted correlation coefficients (*r*) and 95% confidence intervals (95% CI) for the studies organized by first author and year, study number, and the number of extracted parameters of each study. Larger studies were denoted by larger correlation boxes. The meta‐analytic result is represented by a blue diamond.

The analysis examining the association between unpredictability and life history strategy included four articles with 35 extracted correlations and sample sizes (*n =* 4437; see Figure 4 for forest plot). The association was small/medium, negative, and significant (*r =* −0.26, 95% CI = −0.28, −0.24, *p* < 0.0001). A significant (*Q* [34] = 113.68, *p* < 0.0001) and large degree of heterogeneity (*I* ^*2*^ = 70.01%) was identified, with all the heterogeneity attributed between parameters.

## 4 Discussion

The focus of our present analysis was to examine the association between unpredictability and disordered eating. Our four‐level random effects meta‐analysis on 15 articles (83 correlation coefficients; *n* = 9983) found a small, positive association between measures of unpredictability and disordered eating (*r* = 0.12), suggesting a link between stronger perceptions or more experiences of unpredictability and elevated disordered eating in adults and children. Removal of longitudinal associations did not substantially alter the association (*r* = 0.13). Life history theory asserts that individuals who are exposed to harsh and unpredictable environments will develop an elevated unpredictability schema, orienting them toward higher risk behaviors and a faster life history strategy [20, 22]. As such, perceptions and experiences of environmental unpredictability increase obesity risk via unhealthful eating behavior [33], and slower life history strategies should be protective against disordered eating behavior. Accordingly, two additional four‐level random effects meta‐analyses were performed to test theoretically specified linkages and found (1) a small significant negative association between slower life history strategies (*r* = −0.22; nine articles with 46 correlation coefficients, *n* = 9018) and disordered eating, and (2) a small significant negative association between unpredictability and slower life history strategies (*r* = −0.26, four articles with 35 correlation coefficients, *n* = 4437). A prior meta‐analysis of unpredictability examined several outcomes of mental health in adolescents and adults [62]. To our knowledge, this is the first meta‐analysis to systematically review the relationships among unpredictability, life history strategies, and disordered eating.

Our findings are consistent with past meta‐analyses which have found linkages between food insecurity—a domain‐specific aspect of unpredictability—and binge eating [63] and obesity risk [64, 65] (particularly in women). Prior literature on non‐human animal models has also shown that unpredictable access to food, in general, and highly palatable food increase the likelihood of overconsumption [66], may slow energy expenditure [67], and decrease somatic and health maintenance effort [68]. Additionally, the meta‐analytic literature examining childhood maltreatment and adversity has found greater risk of disordered eating [14, 15], particularly with binge eating and bulimia, and obesity [13]. Our findings add to the existing literature by showing that unpredictability, which can occur even without the presence of violence or abuse, elevates one's risk for disordered eating.

Assessment of heterogeneity—defined as variability between studies—is important to assess and may originate from a number of study parameters, including study design characteristics, participant populations, or measures [69]. A large and significant degree of heterogeneity was found regarding the association between unpredictability and disordered eating (*I* ^*2*^ = 86.72%). Moderation analyses found that country of study origin, mode of measurement of disordered eating, chronology of unpredictability, and study percentage of women were significant moderators. Age group status (child vs. adults), and study percentage of white participants were not significant moderators. Although China had a significantly higher correlation coefficient than the United States studies, it is likely that the difference between countries was fueled by different measurement modes used by the countries. For instance, Chinese studies predominately used subjective measures of disordered eating, and most studies with objective measures were from the United States. Although we did not observe a significant moderation when examining the measures as subjective or objective (Table 4), a significant moderation effect was found when all modes of measurement were examined. When compared to self‐report, the snack taste test was significantly different, with a large discrepancy between the coefficient values (0.20 vs. −0.01, respectively). In a sensitivity analysis with both variables (country of origin and mode of measurement) added as moderators, there was no longer a significant difference between China and the USA, but differences between self‐report and the snack taste test remained. Chronology of unpredictability was a significant predictor, suggesting that proximate adult perceptions of unpredictability may be more strongly associated with disordered eating than childhood unpredictability. Although continued investigation of unpredictability across the life‐course is warranted, it is important to note that childhood measures of unpredictability correlate with adult measures [70, 71]. Study percentage of women was a significant moderator, suggesting that sex may be an important factor to consider in future studies. The effect suggests that the larger the percentage of women in the study sample, the stronger the association between unpredictability and disordered eating. However, it is unknown if exposure and experiences of unpredictability differentially impact men and women (see [72] for a brief review suggesting that unpredictability may be more impactful on women).

### 4.1 Limitations and Future Directions

The present meta‐analysis focused on the extraction of unadjusted correlation coefficients that represented the associations between unpredictability and disordered eating. The results of the meta‐analysis were small in magnitude and do not highlight the complexity of the associations and pathways modeled in the emerging literature that may attenuate the direct effect. Thus, we acknowledge that future research should continue to examine theoretically informed mediators, moderators, and confounders that alter the unpredictability–disordered eating association. We did not solicit researchers through research forums or societies, thus potentially excluding relevant unpublished data. Next, we reported results for two additional meta‐analyses that were not the primary study aim. While our systematic search incorporated search terms such as *life history theory* and *life history strategies*, there may be several articles that were omitted in these analyses. Future research should continue to examine differences between age groups and between sexes. Although our age moderator analysis was null, it may be likely that our moderator analyses were underpowered and more studies were needed. Lastly, we included a small subset of articles that comprised an aggregated measure of environmental unpredictability and environmental harshness. Although keeping these constructs separate has been suggested by some researchers [20, 73, 74], we chose to include these measures given the small set of studies and limited previous research with eating behaviors. The inclusion of both measures may have attenuated or biased our associations and is a limitation of the paper.

### 4.2 Recommendations

Based on the results of the systematic review and meta‐analysis, we offer recommendations to move the burgeoning field of unpredictability and disordered eating forward. Foremost, incorporation of measures of adiposity (e.g., weight, BMI, body fat) or appetitive biomarkers and physiology surrogates into the larger nomological networks [75] to inform statistical models should be a priority. A large subset of articles surveyed did not have these measures. Researchers should continue to incorporate multiple measures of disordered eating (e.g., self‐report, objective measures), prioritizing objective measures to continue to examine whether the small subset of studies with objective measures were underpowered. Additional investigators should include multiple measures of unpredictability experiences as well an assessment of an unpredictability schema. There are many subfields in psychology that are broadly examining the importance of experiences, exposures, and perceptions of unpredictability; however, these subfields appear to be agnostic to the measures the other fields are using (e.g., disordered eating researchers do not use the Questionnaire of Unpredictability in Childhood (QUIC [76]); neuropsychologists do not use the Scale of Unpredictability Beliefs (SUB [70])). Capitalizing on these multiple measures will improve measurement precision and will facilitate the use of more advanced statistics that model latent variables, such as structural equation modeling. In addition, only a small subset of studies had longitudinal associations. Future studies should continue to examine the differences between adults and children and similarly assess whether the effect of unpredictability persists through the lifespan. Sex differences should also be considered. Finally, psychometricians and clinicians should work on creating clinically‐relevant cut‐offs for existing measures of unpredictability measures and/or design measures that may potentially inform interventions while being careful to not “*declaw the cat*” [77, 78] (i.e., “removing the psychological and behavioral weaponry necessary to survive and control resources in one's local ecology,” p. 610 [77]).

## 5 Conclusion

Given the association between disordered eating behavior and obesity, our findings from this systematic review provide evidence to consider the role of unpredictability in human development and its consequences in disordered eating. In accordance with previous findings, this work suggests that addressing obesity risk may be multifactorial. Environmental‐level policy changes promoting predictability and stability at the neighborhood or household level may potentially diminish experiences and perceptions of unpredictability, promoting increased health‐maintaining behaviors, including more salubrious eating. At the group and individual level, developing models that measure and address degrees of unpredictability could be helpful in the development of successful dietary interventions.

## Author Contributions

Conceptualization: Tomás Cabeza de Baca; Methodology: Tomás Cabeza de Baca, Hannah T. Fry, Gisela Butera; Software: Tomás Cabeza de Baca; Formal analysis and investigation: Tomás Cabeza de Baca, Hannah T. Fry, Brooke Bitsuie; Data Curation: Hannah T. Fry, Tomás Cabeza de Baca, Brooke Bitsuie; Visualization: Tomás Cabeza de Baca, Hannah T. Fry; Writing – original draft preparation: Tomás Cabeza de Baca, Hannah T. Fry, Andrés M. Treviño Alvarez; Writing – review and editing: Tomás Cabeza de Baca, Hannah T. Fry, Andrés M. Treviño Alvarez, Gisela Butera, Brooke Bitsuie, Marci E. Gluck; Supervision: Marci E. Gluck.

## Conflicts of Interest

The authors declare no conflicts of interest.

## Supporting information


**Data S1:** Supporting Information.

## Data Availability

The data that support the findings of this study are available from the corresponding author upon reasonable request.
