# Supplementary material for: A Systematic Review and Meta‐Analytic Assessment of Unpredictability and Disordered Eating
Source: Obes Rev. 2025 Oct 1;27(2):e70022. doi: 10.1111/obr.70022 (PMC12812503; doi:10.1111/obr.70022)

A Systematic Review and Meta-Analytic Assessment of Unpredictability and Disordered Eating

Tomás Cabeza de Baca[1], Hannah T. Fry[1,2], Andrés M. Treviño-Alvarez[1,3], Gisela Butera[5], Brooke Betsuie[4], Marci E. Gluck[1]

[1]: Obesity and Diabetes Clinical Research Section, Phoenix Epidemiology and Clinical Research Branch, National Institute of Diabetes and Digestive and Kidney Diseases, Phoenix, Arizona, USA

[2]: Warren Alpert Medical School of Brown University, Providence, Rhode Island, USA

[3]: Department of Neurology, Universidad Autonoma de Nuevo Leon, Monterrey, México

[4]: Department of Health Sciences, College of Health and Human Services, Northern Arizona University, Flagstaff, Arizona, USA

[5]: Office of Research Services, Division of Library Services, National Institutes of Health Library, Bethesda, Maryland, USA

Running Title: Unpredictability Meta-Analysis

Corresponding Author: Tomás Cabeza de Baca, PhD, Obesity and Diabetes Clinical Research Section, Phoenix Epidemiology and Clinical Research Branch, National Institute of Diabetes and Digestive and Kidney Diseases, National Institutes of Health, 4212 North 16th Street, Phoenix, Arizona, 85016, USA.

E-mail Address: tommy.cabezadebaca@nih.gov

NIH National Institutes of Health
Office of Management

Supplemental Table S1. Database Search Strategies

Database: PubMed/MEDLINE
Platform: National Library of Medicine
Date Searched: 7/18/2023
Limits: Human studies (exclude: editorials, letters, and retractions)

	Concept:	Search Strategy:
#1	Disordered Eating	"disordered eating"[Title/Abstract] OR "emotional eat*"[Title/Abstract] OR "Food Addiction"[Mesh] OR "food addict*"[Title/Abstract] OR "food addiction"[Title/Abstract:~4] OR "food responsiv*"[Title/Abstract] OR "food cue responsiv*"[Title/Abstract] OR "bored eating"[Title/Abstract:~4] OR "compulsive eat*"[Title/Abstract] OR "disinhibited eat*"[Title/Abstract] OR "stress eating"[Title/Abstract:~4] OR "Night Eating Syndrome"[Mesh] OR "night eating"[Title/Abstract:~4] OR "food enjoyment"[Title/Abstract:~4] OR "Taste Perception"[Mesh] OR "taste perception"[Title/Abstract] OR "taste test"[Title/Abstract:~2] OR "Portion Size"[Mesh] OR "portion size"[Title/Abstract:~2] OR "food portion*"[Title/Abstract] OR "meal portion*"[Title/Abstract] OR "overeat*"[Title/Abstract] OR "over eat*"[Title/Abstract] OR "calories consum*"[Title/Abstract] OR "mindful eat*"[Title/Abstract] OR "mindless eat*"[Title/Abstract] OR "obesity risk*"[Title/Abstract] OR "risk of obesity"[Title/Abstract] OR "Binge-Eating Disorder"[Mesh] OR "binge eat*"[Title/Abstract] OR "Hunger"[Mesh] OR "hunger"[Title/Abstract] OR "Satiation"[Mesh] OR "satiat*"[Title/Abstract] OR "Palatable Eating Motives Scale"[Title/Abstract] OR "Three Factor Eating Questionnaire"[Title/Abstract] OR "TFEQ"[Title/Abstract] OR (("Food"[Mesh] OR "food*"[Title/Abstract] OR "Eating"[Mesh] OR "eating*"[Title/Abstract]) AND ("loss of control"[Title/Abstract] OR "restraint*"[Title/Abstract] OR "over consum*"[Title/Abstract] OR "overconsume*"[Title/Abstract] OR "hedoni*"[Title/Abstract]))
#2	Unpredictability Schema	"unpredictab*"[Title/Abstract] OR "Resilience, Psychological"[Mesh] OR "resilienc*"[Title/Abstract] OR "Life Course Perspective"[Mesh] OR "Life History Traits"[Mesh] OR "life histor*"[Title/Abstract] OR "Body Image Questionnaire"[Title/Abstract] OR "Questionari d'Imatge Corporal"[Title/Abstract:~0] OR "QUIC"[Title/Abstract] OR "Confusion, Hubbub, and Order Scale"[Title/Abstract:~0] OR "CHAOS scale"[Title/Abstract:~4] OR "stress scale"[Title/Abstract] OR "job change*"[Title/Abstract] OR "Social Support"[Mesh] OR "social support*"[Title/Abstract] OR "family support*"[Title/Abstract] OR ("transition*"[Title/Abstract] AND ("Parents"[Mesh] OR "parent*"[Title/Abstract])) OR "Home Environment"[Mesh] OR "home environment*"[Title/Abstract] OR "residential change*"[Title/Abstract] OR "caregiver chang*"[Title/Abstract] OR "Housing Instability"[Mesh] OR "housing instabilit*"[Title/Abstract] OR "housing stabilit*"[Title/Abstract] OR "housing insecurit*"[Title/Abstract] OR "housing securit*"[Title/Abstract] OR "number moves"[Title/Abstract:~4] OR (("Death"[Mesh] OR "death*"[Title/Abstract] OR "Bereavement"[Mesh] OR "bereave*"[Title/Abstract] OR "grief"[Title/Abstract]) AND ("Family"[Mesh] OR "famil*"[Title/Abstract] OR "parent*"[Title/Abstract]

		OR "mother*"[Title/Abstract] OR "father*"[Title/Abstract] OR "spouse*"[Title/Abstract] OR "Caregivers"[Mesh] OR "caregiver*"[Title/Abstract] OR "care giver*"[Title/Abstract] OR "Legal Guardians"[Mesh] OR "guardian*"[Title/Abstract])) OR "Unemployment"[Mesh] OR "unemploy*"[Title/Abstract] OR "job insecurity"[Title/Abstract] OR "job loss"[Title/Abstract:~2] OR "loss of employment"[Title/Abstract] OR "income loss"[Title/Abstract:~2]
#3	Limits & Filters	((#1 AND #2) NOT ("Animals"[Mesh] NOT ("Animals"[Mesh] AND "Humans"[Mesh]))) NOT (("Editorial" [Publication Type] OR "Comment" [Publication Type] OR "Letter" [Publication Type] OR retracted publication[Publication Type] OR retraction of publication[Publication Type] OR "retraction of publication*"[Title/Abstract] OR "retraction notice"[Title] OR "retracted publication"[Title])

Database: Cochrane Library
Platform: Wiley & Sons
Date Searched: 7/18/2023
Limits: Human studies (exclude: editorials, letters, and retractions)

	Concept:	Search Strategy:
#1	Disordered Eating	("disordered eating" OR (emotional NEXT/2 eat*) OR [mh "Food Addiction"] OR (food NEXT/2 addict*) OR (food NEAR/4 addiction) OR (food NEXT/2 responsiv*) OR ("food cue" NEXT/2 responsiv*) OR (bored NEAR/4 eating) OR (compulsive NEXT/2 eat*) OR (disinhibited NEXT/2 eat*) OR (stress NEAR/4 eating) OR [mh "Night Eating Syndrome"] OR (night NEAR/4 eating) OR (food NEAR/4 enjoyment) OR [mh "Taste Perception"] OR "taste perception" OR (taste NEAR/2 test) OR [mh "Portion Size"] OR (portion NEAR/2 size) OR (food NEXT/2 portion*) OR (meal NEXT/2 portion*) OR overeat* OR (over NEXT/2 eat*) OR (calories NEXT/2 consum*) OR (mindful NEXT/2 eat*) OR (mindless NEXT/2 eat*) OR (obesity NEXT/2 risk*) OR "risk of obesity" OR [mh "Binge-Eating Disorder"] OR (binge NEXT/2 eat*) OR [mh "Hunger"] OR hunger OR [mh "Satiation"] OR satiat* OR "Palatable Eating Motives Scale" OR "Three Factor Eating Questionnaire" OR "TFEQ" OR (([mh "Food"] OR food* OR [mh "Eating"] OR eating*) AND ("loss of control" OR restraint* OR (over NEXT/2 consum*) OR overconsume* OR hedoni*))):ti,ab,kw
#2	Unpredictability Schema	unpredictab* OR [mh "Resilience, Psychological"] OR resilienc* OR [mh "Life Course Perspective"] OR [mh "Life History Traits"] OR (life NEXT/2 histor*) OR "Body Image Questionnaire" OR "Questionari d'Imatge Corporal" OR "QUIC" OR "Confusion, Hubbub, and Order Scale" OR (CHAOS NEAR/4 scale) OR "stress scale" OR (job NEXT/2 change*) OR [mh "Social Support"] OR (social NEXT/2 support*) OR (family NEXT/2 support*) OR (transition* NEAR/4 ([mh "Parents"] OR parent*)) OR [mh "Home Environment"] OR (home NEXT/2 environment*) OR (residential NEXT/2 change*) OR (caregiver NEXT/2 chang*) OR [mh "Housing Instability"] OR (housing NEXT/2 instabilit*) OR (housing NEXT/2

		stabilit*) OR (housing NEXT/2 insecurit*) OR (housing NEXT/2 securit*) OR (number NEAR/4 moves) OR [mh "Unemployment"] OR unemploy* OR "job insecurity" OR (job NEAR/2 loss) OR "loss of employment" OR (income NEAR/2 loss) OR (([mh "Death"] OR death* OR [mh "Bereavement"] OR bereave* OR grief) NEAR/4 ([mh "Family"] OR famil* OR parent* OR mother* OR father* OR spouse* OR [mh "Caregivers"] OR caregiver* OR (care NEXT/2 giver*) OR [mh "Legal Guardians"] OR guardian*))
#3	Limits & Filters	#1 AND #2" (Word variations have been searched)

Database: Embase
Platform: Elsevier
Date Searched: 7/18/2023
Limits: Human studies (exclude: editorials, letters, and retractions)

	Concept:	Search Strategy:
#1	Disordered Eating	'food addiction'/exp OR 'night eating syndrome'/exp OR 'taste'/exp/mj OR 'portion size'/exp OR 'binge eating disorder'/exp OR 'hunger'/exp OR 'satiety'/exp OR 'disordered eating':ab,ti OR 'emotional eat*':ab,ti OR 'food addict*':ab,ti OR ((food NEAR/4 addiction*):ab,ti) OR 'food responsiv*':ab,ti OR 'food cue responsiv*':ab,ti OR ((bored NEAR/4 eating):ab,ti) OR 'compulsive eat*':ab,ti OR 'disinhibited eat*':ab,ti OR ((stress NEAR/4 eating):ab,ti) OR ((night NEAR/4 eating):ab,ti) OR ((food NEAR/4 enjoyment):ab,ti) OR 'taste perception':ab,ti OR ((taste NEAR/2 test):ab,ti) OR ((portion NEAR/2 size):ab,ti) OR 'food portion*':ab,ti OR 'meal portion*':ab,ti OR 'overeat*':ab,ti OR 'over eat*':ab,ti OR 'calories consum*':ab,ti OR 'mindful eat*':ab,ti OR 'mindless eat*':ab,ti OR 'obesity risk*':ab,ti OR 'risk of obesity':ab,ti OR 'binge eat*':ab,ti OR 'hunger':ab,ti OR 'satiat*':ab,ti OR 'palatable eating motives scale':ab,ti OR 'three factor eating questionnaire':ab,ti OR 'tfeq':ab,ti OR (('food*':ab,ti OR 'eating*':ab,ti) AND ('loss of control':ab,ti OR 'restraint*':ab,ti OR 'over consum*':ab,ti OR 'overconsume*':ab,ti OR 'hedoni*':ab,ti))
#2	Unpredictability Schema	'psychological resilience'/exp OR 'life course perspective'/exp OR 'life history trait'/exp OR 'social support'/exp OR 'home environment'/exp/mj OR 'housing instability'/exp OR 'unemployment'/exp OR unpredictab*:ab,ti OR resilienc*:ab,ti OR 'life histor*':ab,ti OR 'body image questionnaire':ab,ti OR ((questionari NEAR/2 corporal):ab,ti) OR 'quic':ab,ti OR (('confusion, hubbub' NEAR/2 'order scale'):ab,ti) OR ((chaos NEAR/4 scale):ab,ti) OR 'stress scale':ab,ti OR 'job change*':ab,ti OR 'social support*':ab,ti OR 'family support*':ab,ti OR ((transition* NEAR/4 parent*):ab,ti) OR 'home environment*':ab,ti OR 'residential change*':ab,ti OR 'caregiver chang*':ab,ti OR 'housing instabilit*':ab,ti OR 'housing stabilit*':ab,ti OR ((job NEAR/2 loss):ab,ti) OR 'loss of employment':ab,ti OR ((income NEAR/2 loss):ab,ti) OR 'housing insecurit*':ab,ti OR 'housing securit*':ab,ti OR ((number NEAR/4 moves):ab,ti) OR unemploy*:ab,ti OR 'job insecurity':ab,ti OR (((death* OR bereave* OR grief) NEAR/4

		(famil* OR parent* OR mother* OR father* OR spouse* OR caregiver* OR 'care giver*' OR guardian*)):ab,ti)
#3	Limits & Filters	#1 AND #2 NOT ([animals]/lim NOT ([animals]/lim AND [humans]/lim)) NOT ([editorial]/lim OR [letter]/lim OR [note]/lim OR 'retraction of publication':ab,ti OR 'retraction notice':ti OR 'retracted publication':ab,ti) AND ([article]/lim OR [article in press]/lim OR [review]/lim)

Database: Web of Science (Core Collection)
Platform: Clarivate Analytics
Date Searched: 7/18/2023
Limits: Human studies (exclude: editorials, letters, and retractions)

	Concept:	Search Strategy:
#1	Disordered Eating	"disordered eating" OR "emotional eat*" OR "food addict*" OR (food NEAR/4 addiction) OR "food responsiv*" OR "food cue responsiv*" OR (bored NEAR/4 eating) OR "compulsive eat*" OR "disinhibited eat*" OR (stress NEAR/4 eating) OR (night NEAR/4 eating) OR (food NEAR/4 enjoyment) OR "taste perception" OR (taste NEAR/2 test) OR (portion NEAR/2 size) OR "food portion*" OR "meal portion*" OR overeat* OR "over eat*" OR "calories consum*" OR "mindful eat*" OR "mindless eat*" OR "obesity risk*" OR "risk of obesity" OR "binge eat*" OR hunger OR satiat* OR "Palatable Eating Motives Scale" OR "Three Factor Eating Questionnaire" OR "TFEQ" OR ((food* OR eating*) AND ("loss of control" OR restraint* OR "over consum*" OR overconsume* OR hedoni*))
#2	Unpredictability Schema	unpredictab* OR resilienc* OR "life histor*" OR "Body Image Questionnaire" OR "Questionari d'Imatge Corporal" OR "QUIC" OR "Confusion, Hubbub, and Order Scale" OR (CHAOS NEAR/4 scale) OR "stress scale" OR "job change*" OR "social support*" OR "family support*" OR (transition* NEAR/4 parent*) OR "home environment*" OR "residential change*" OR "caregiver chang*" OR "housing instabilit*" OR "housing stabilit*" OR "housing insecurit*" OR "housing securit*" OR (number NEAR/4 moves) OR unemploy* OR "job insecurity" OR (job NEAR/2 loss) OR "loss of employment" OR (income NEAR/2 loss) OR ((death* OR bereave* OR grief) NEAR/4 (famil* OR parent* OR mother* OR father* OR spouse* OR caregiver* OR "care giver*" OR guardian*))
#3	Limits & Filters	#2 AND #1 and Preprint Citation Index (Exclude – Database) and Web of Science Core Collection (Database) and Animals (Exclude – MeSH Headings) and Humans (MeSH Headings) and Letter or Editorial Material or Retracted Publication or Retraction (Exclude – Document Types) and Article or Review Article or Meeting or Abstract (Document Types)

Database: PsycInfo
Platform: American Psychological Association
Date Searched: 7/18/2023
Limits: Human studies (exclude: editorials, letters, and retractions)

	Concept:	Search Strategy:
	Disordered Eating AND Unpredictability Schema Limits and Filters: Human and Peer Review	((IndexTermsFilt: ("Resilience (Psychological)") OR IndexTermsFilt: ("Life Span") OR IndexTermsFilt: ("Life Experiences") OR IndexTermsFilt: ("Social Support") OR IndexTermsFilt: ("Home Environment") OR IndexTermsFilt: ("Unemployment")) OR (title: (unpredictab*) OR title: (resilienc*) OR title: ("life histor*") OR title: ("Body Image Questionnaire") OR title: ("Questionari d'Imatge Corporal") OR title: ("QUIC") OR title: ("Confusion, Hubbub, and Order Scale") OR (title: (CHAOS NEAR/4 scale)) OR title: ("stress scale") OR title: ("job change*") OR title: ("social support*") OR title: ("family support*") OR (title: (transition* NEAR/4 parent*)) OR title: ("home environment*") OR title: ("residential change*") OR title: ("caregiver chang*") OR title: ("housing instabilit*") OR title: ("housing stabilit*") OR title: ("housing insecurit*") OR title: ("housing securit*") OR (title: (number NEAR/4 moves)) OR title: (unemploy*) OR title: ("job insecurity") OR (title: (job NEAR/2 loss)) OR title: ("loss of employment") OR (title: (income NEAR/2 loss)) OR ((title: (bereave*) OR title: (grief)) NEAR/4 (title: (famil*) OR title: (parent*) OR title: (mother*) OR title: (father*) OR title: (spouse*) OR title: (caregiver*) OR title: ("care giver*") OR title: (guardian*)))) OR (abstract: (unpredictab*) OR abstract: (resilienc*) OR abstract: ("life histor*") OR abstract: ("Body Image Questionnaire") OR abstract: ("Questionari d'Imatge Corporal") OR abstract: ("QUIC") OR abstract: ("Confusion, Hubbub, and Order Scale") OR (abstract: (CHAOS NEAR/4 scale)) OR abstract: ("stress scale") OR abstract: ("job change*") OR abstract: ("social support*") OR abstract: ("family support*") OR (abstract: (transition* NEAR/4 parent*)) OR abstract: ("home environment*") OR abstract: ("residential change*") OR abstract: ("caregiver chang*") OR abstract: ("housing instabilit*") OR abstract: ("housing stabilit*") OR abstract: ("housing insecurit*") OR abstract: ("housing securit*") OR (abstract: (number NEAR/4 moves)) OR abstract: (unemploy*) OR abstract: ("job insecurity") OR (abstract: (job NEAR/2 loss)) OR abstract: ("loss of employment") OR (abstract: (income NEAR/2 loss)) OR ((abstract: (bereave*) OR abstract: (grief)) NEAR/4 (abstract: (famil*) OR abstract: (parent*) OR abstract: (mother*) OR abstract: (father*) OR abstract: (spouse*) OR abstract: (caregiver*) OR abstract: ("care giver*") OR abstract: (guardian*))))) AND ((IndexTermsFilt: ("Food Addiction") OR IndexTermsFilt: ("Taste Perception") OR IndexTermsFilt: ("Binge Eating Disorder") OR IndexTermsFilt: ("Hunger") OR IndexTermsFilt: ("Satiation")) OR (title: ("disordered eating") OR title: ("emotional eat*") OR title: ("food addict*") OR (title: (food NEAR/4 addiction)) OR title: ("food responsiv*") OR title: ("food cue responsiv*") OR (title: (bored NEAR/4 eating)) OR title: ("compulsive eat*") OR title: ("disinhibited eat*") OR (title: (stress NEAR/4 eating)) OR (title: (night NEAR/4 eating)) OR (title: (food

		NEAR/4 enjoyment)) OR title: ("taste perception") OR (title: (taste NEAR/2 test)) OR (title: (portion NEAR/2 size)) OR title: ("food portion*") OR title: ("meal portion*") OR title: (overeat*) OR title: ("over eat*") OR title: ("calories consum*") OR title: ("mindful eat*") OR title: ("mindless eat*") OR title: ("obesity risk*") OR title: ("risk of obesity") OR title: ("binge eat*") OR title: (hunger) OR title: (satiat*) OR title: ("Palatable Eating Motives Scale") OR title: ("Three Factor Eating Questionnaire") OR title: ("TFEQ") OR ((title: (food*) OR title: (eating*)) AND (title: ("loss of control") OR title: (restraint*) OR title: ("over consum*") OR title: (overconsume*) OR title: (hedoni*)))) OR (abstract: ("disordered eating") OR abstract: ("emotional eat*") OR abstract: ("food addict*") OR (abstract: (food NEAR/4 addiction)) OR abstract: ("food responsiv*") OR abstract: ("food cue responsiv*") OR (abstract: (bored NEAR/4 eating)) OR abstract: ("compulsive eat*") OR abstract: ("disinhibited eat*") OR (abstract: (stress NEAR/4 eating)) OR (abstract: (night NEAR/4 eating)) OR (abstract: (food NEAR/4 enjoyment)) OR abstract: ("taste perception") OR (abstract: (taste NEAR/2 test)) OR (abstract: (portion NEAR/2 size)) OR abstract: ("food portion*") OR abstract: ("meal portion*") OR abstract: (overeat*) OR abstract: ("over eat*") OR abstract: ("calories consum*") OR abstract: ("mindful eat*") OR abstract: ("mindless eat*") OR abstract: ("obesity risk*") OR abstract: ("risk of obesity") OR abstract: ("binge eat*") OR abstract: (hunger) OR abstract: (satiat*) OR abstract: ("Palatable Eating Motives Scale") OR abstract: ("Three Factor Eating Questionnaire") OR abstract: ("TFEQ") OR ((abstract: (food*) OR abstract: (eating*)) AND (abstract: ("loss of control") OR abstract: (restraint*) OR abstract: ("over consum*") OR abstract: (overconsume*) OR abstract: (hedoni*))))) AND NOT Population Group: Animal AND Population Group: Human AND Peer-Reviewed Journals only

Database: Dissertation & Thesis
Platform: ProQuest
Date Searched: 7/18/2023
Limits: Human studies & Dissertations/Thesis

	Concept:	Search Strategy:
#1	Disordered Eating AND Unpredictability Schema Limits and Filters: Dissertation & Thesis	su("disordered eating" OR "emotional eat*" OR "food addict*" OR (food NEAR/4 addiction) OR "food responsiv*" OR "food cue responsiv*" OR (bored NEAR/4 eating) OR "compulsive eat*" OR "disinhibited eat*" OR (stress NEAR/4 eating) OR (night NEAR/4 eating) OR (food NEAR/4 enjoyment) OR "taste perception" OR (taste NEAR/2 test) OR (portion NEAR/2 size) OR "food portion*" OR "meal portion*" OR overeat* OR "over eat*" OR "calories consum*" OR "mindful eat*" OR "mindless eat*" OR "obesity risk*" OR "risk of obesity" OR "binge eat*" OR hunger OR satiat* OR "Palatable Eating Motives Scale" OR "Three Factor Eating Questionnaire" OR "TFEQ" OR ((food* OR eating*) AND ("loss of control" OR restraint* OR "over consum*" OR overconsume* OR hedoni*))) AND su(unpredictab* OR resilienc* OR "life histor*" OR "Body Image Questionnaire"

		OR "Questionari d'Imatge Corporal" OR "QUIC" OR "Confusion, Hubbub, and Order Scale" OR (CHAOS NEAR/4 scale) OR "stress scale" OR "job change*" OR "social support*" OR "family support*" OR (transition* NEAR/4 parent*) OR "home environment*" OR "residential change*" OR "caregiver chang*" OR "housing instabilit*" OR "housing stabilit*" OR "housing insecurit*" OR "housing securit*" OR (number NEAR/4 moves) OR unemploy* OR "job insecurity" OR (job NEAR/2 loss) OR "loss of employment" OR (income NEAR/2 loss) OR ((death* OR bereave* OR grief) NEAR/4 (famil* OR parent* OR mother* OR father* OR spouse* OR caregiver* OR "care giver*" OR guardian*))) Limited: Dissertations & Theses

Supplemental Document S2. Newcastle-Ottawa Scale adapted for cross-sectional studies.

Selection:

SELECTION 1

1. Representativeness of the sample:
   a. Truly representative of the average in the target population. * (all subjects or random sampling)
   b. Somewhat representative of the average in the target group. * (non-random sampling)
   c. Selected group of users/convenience sample.
   d. No description of the sampling strategy.

SELECTION 2

2. Sample size:
   a. Justified and satisfactory (including sample size calculation). *
   b. Not justified/ no information provided

SELECTION 3

3. Non-respondents:
   a. Proportion of target sample recruited attains pre-specified target or basic summary of non-respondent characteristics in sampling frame recorded. *
   b. Unsatisfactory recruitment rate, no summary data on non-respondents or missing data.
   c. No information provided

SELECTION 4

4. Ascertainment of validation (Unpredictability) (or life history strategy if no unpredictability):
   a. Validated measurement tool. **
   b. Non-validated measurement tool, but is available or described. *
   c. No description of the measurement tool.

Comparability: (Maximum 2 stars)

COMPARABILITY 1

1. Comparability of subjects in different outcome groups on the basis of design or analysis. Confounding factors controlled.
   a. Data/ results adjusted or reported for relevant predictors/risk factors/confounders e.g., age, sex, time since vaccination, etc. **
   b. Data/results not adjusted or reported for all relevant confounders/risk factors/information not provided.

Outcome:

OUTCOME1

1. Assessment of outcome (disordered eating behavior):
   a. Validated clinician interview tool or objective task. **
   b. Validated measurement tool **
   c. non-validated measurement tool, but is available or described. *
   d. No description of measurement tool.

OUTCOME2

2. Statistical test:
   a. Statistical test used to analyse the data clearly described, appropriate and measures of association presented including confidence intervals and probability level (p value). *
   b. Statistical test not appropriate, not described or incomplete.

Cross-sectional Studies:
Very Good Studies: 9-10 points
Good Studies: 7-8 points
Satisfactory Studies: 5-6 points
Unsatisfactory Studies: 0 to 4 points

This scale has been adapted from the Newcastle-Ottawa Quality Assessment Scale for cohort studies to provide quality assessment of cross sectional studies[1].

---

[1] Herzog R, et al. Is Healthcare Workers' Intention to Vaccinate Related to their Knowledge, Beliefs and Attitudes? A Systematic Review. *BMC Public Health* 2013 **13**:154

Patra, J., Bhatia, M., Suraweera, W., Morris, S. K., Patra, C., Gupta, P. C., & Jha, P. (2015). Exposure to second-hand smoke and the risk of tuberculosis in children and adults: a systematic review and meta-analysis of 18 observational studies. PLoS medicine, 12(6), e1001835.

Supplemental Table S4. Excluded Articles List

	Excluded Article:	Exclusion reason:
1	Buchanan GJR, Tate AD, Loth KA, Trofholz AC, Berge JM. CHAOS in the Home Environment and Child Weight-Related Outcomes. J Am Board Fam Med. 2021;34(6):1163-1173. doi:10.3122/jabfm.2021.06.210157	Extractable data unavailable to calculate effect size;
2	Gonçalves S, Moreira C, Gonçalves M, Vieira AI, Machado BC. The role of the perception of family environment in relation to body dissatisfaction, disordered eating and difficulties in close relationships. Eating and weight disorders. 2020;25(1):205-213. doi:10.1007/s40519-018-0551-9	No measure of unpredictability
3	Haslam M, Mountford V, Meyer C, Waller G. Invalidating childhood environments in anorexia and bulimia nervosa. Eating Behaviors. 2008;9(3):313-318. doi:10.1016/j.eatbeh.2007.10.005	No measure of unpredictability
4	Hill SE, Prokosch ML, DelPriore DJ, Griskevicius V, Kramer A. Low Childhood Socioeconomic Status Promotes Eating in the Absence of Energy Need. Psychological Science. 2016;27(3):354-364. doi:10.1177/0956797615621901	No measure of unpredictability
5	Laran J, Salerno A. Life-History Strategy, Food Choice, and Caloric Consumption. Psychological Science. 2013;24(2):167-173. doi:10.1177/0956797612450033	No measure of unpredictability
6	Leung CY, Miller AL, Kaciroti NA, Chen YP, Rosenblum K, Lumeng JC. Low-income pre-schoolers with higher temperamental surgency enjoy and respond more to food, mediating the path to higher body mass index. Pediatr Obes. 2016;11(3):181-6. doi:10.1111/ijpo.12042	Cohort overlap with another publication;
7	Proffitt Leyva RP, Hill SE. The impact of childhood unpredictability, interoceptive body awareness, and blood glucose on eating behavior. 2016;(10246515)	Thesis/Dissertation overlap with Publication

8	Quick V, Martin-Biggers J, Povis GA, Hongu N, Worobey J, Byrd-Bredbenner C. A Socio-Ecological Examination of Weight-Related Characteristics of the Home Environment and Lifestyles of Households with Young Children. Nutrients. 2017;9(6)doi:10.3390/nu9060604	No measure of disordered eating
9	Riley HO, Lo SL, Rosenblum K, et al. Sex differences in the association between household chaos and body mass index z-score in low-income toddlers. Childhood Obesity. 2020;16(4):265-273. doi:10.1089/chi.2019.0186	No measure of disordered eating
10	Zhu H, Luo X, Cai T, He J, Lu Y, Wu S. Life event stress and binge eating among adolescents: The roles of early maladaptive schemas and impulsivity. Stress and Health: Journal of the International Society for the Investigation of Stress. 2016;32(4):395-401. doi:10.1002/smi.2634	No measure of unpredictability

Supplemental Figure S4. Funnel Plot

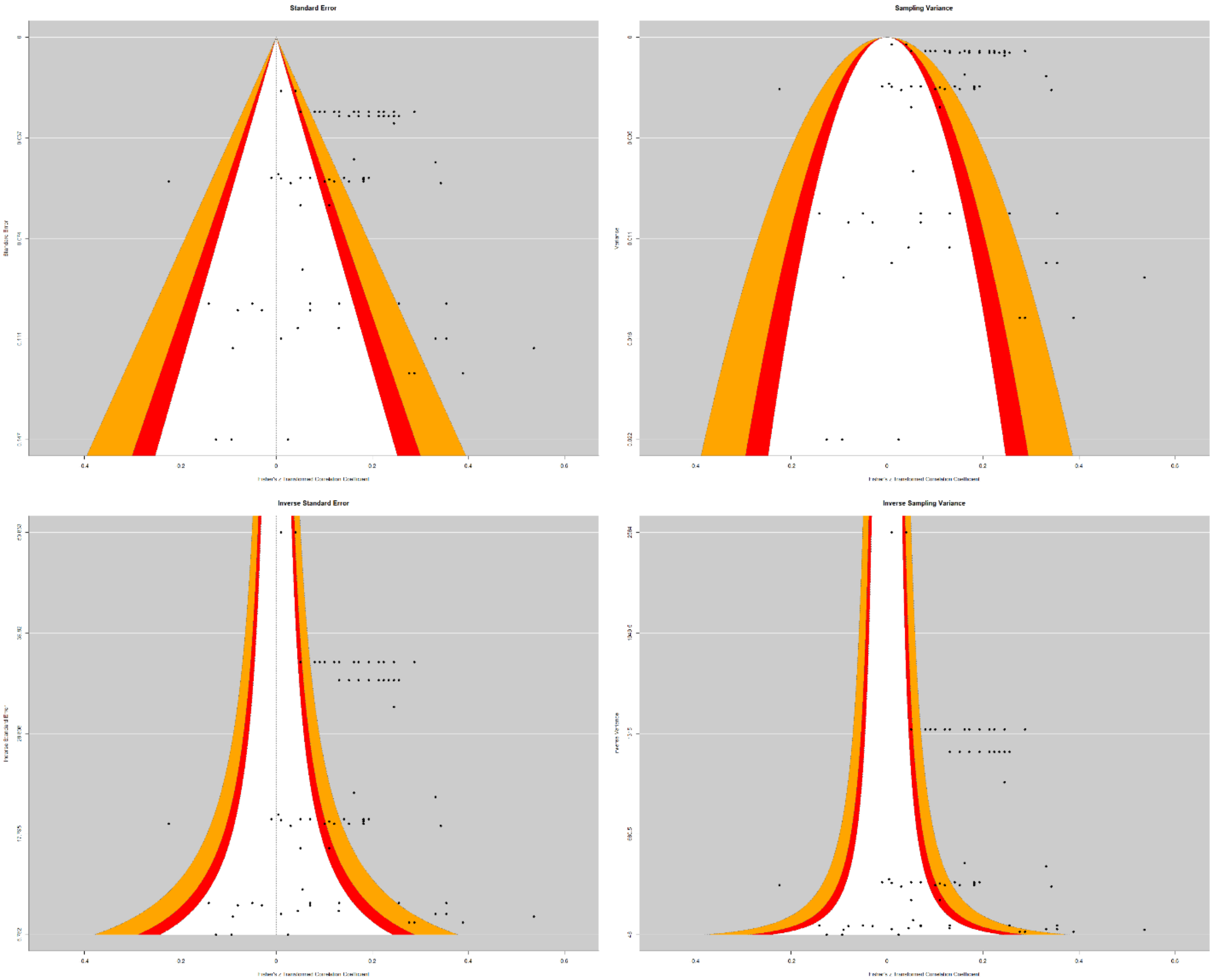

Supplement: Supplementary file 1 — Data S1: Supporting Information. [file OBR-27-e70022-s001.pdf]
